# Classic Kaposi’s sarcoma with an initial solitary colon lesion

**DOI:** 10.1002/ccr3.2080

**Published:** 2019-02-27

**Authors:** Guido Carillio, Luigi Tucci, Stefano Molica

**Affiliations:** ^1^ Department of Oncology and Hematology “Pugliese‐Ciaccio” Hospital Catanzaro Italy; ^2^ Department of Pathological Anatomy “Pugliese‐Ciaccio” Hospital Catanzaro Italy

**Keywords:** classic Kaposi’s sarcoma, HHV‐8 positive colon lesion

## Abstract

Kaposi's sarcoma involving the digestive tract in isolated form before the appearance of diffuse skin lesions is very rare in HIV‐negative patients and is a condition requiring watchful waiting.

We describe a case of a 61‐year‐old woman undergoing an excision of a solitary nodule in the colon, with diagnosis of classic Kaposi's sarcoma. Cutaneous lesions appeared on the legs after three months from colon manifestation. Kaposi's sarcoma involving the digestive tract before skin lesions is very rare in HIV‐negative patients.

## 1 QUESTION

What is the percentage of Kaposi's sarcoma involving the digestive tract in HIV‐negative patients?

## 2 DISCUSSION

**Figure 1:**
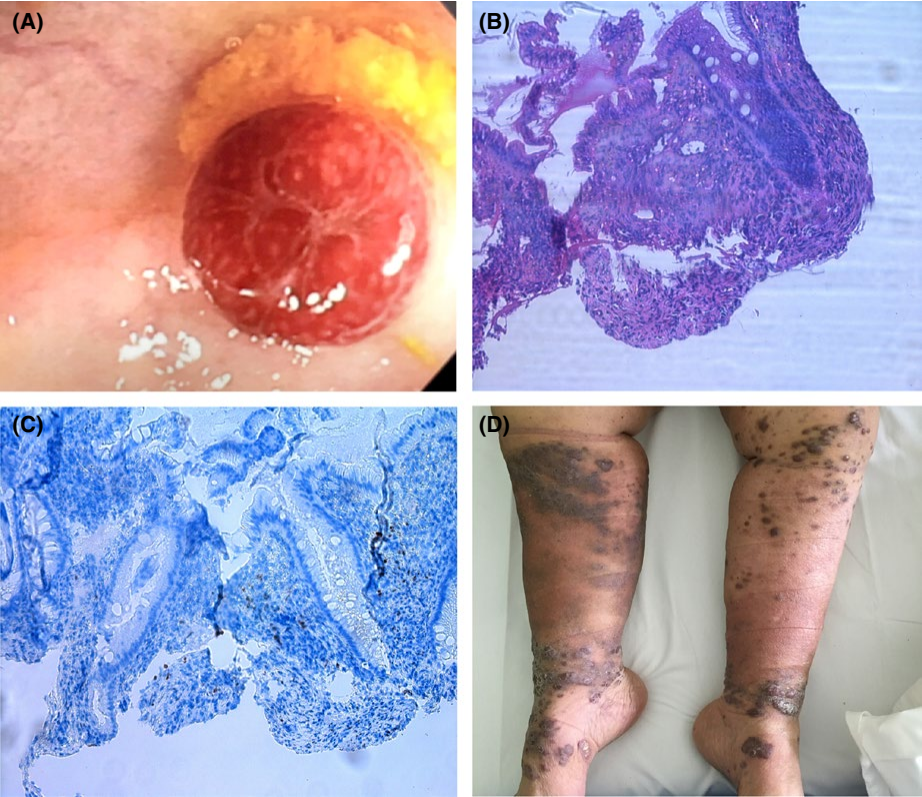
solitary nodule of Kaposi's sarcoma in the transverse colon (A); diagnosis by histology (B) and HHV‐8 immunostaining (C); skin nodules of Kaposi's sarcoma on the legs (D)

A 61‐year‐old woman was admitted to our hospital due to Stevens‐Johnson's reaction of uncertain etiology and mild anemia. Diffuse cutaneous macule‐papules were observed in thorax and lower limbs and empirically treated with antibiotics and steroids. Endoscopic examinations of stomach were negative, while colonoscopy allowed to evidence and remove a solitary purple nodule in the transverse colon, with diagnosis of classic Kaposi's sarcoma (Figure 1A,B) positive for human herpesvirus‐8 (Figure 1C). HIV test was negative. A watchful follow‐up was proposed. After three months, gross skin lesions appeared on the legs (Figure 1D), and a cutaneous biopsy permitted to diagnose a dermal Kaposi's sarcoma in nodular phase. Thus, the patient was sent to the oncology department. Kaposi's sarcoma presenting in the gastrointestinal tract before skin involvement affects only 10% of HIV‐negative patients.^1^, ^2^ CT scan ruled out other visible localizations. Chemotherapy with pegylated liposomal doxorubicin at 20 mg/m^2^ every two weeks yielded partial response and was effectively continued until 12 courses.

## CONFLICT OF INTEREST

None declared.

## AUTHOR CONTRIBUTION

GC: participated in the management of patient care and wrote the paper. LT: analyzed the colon tissue specimens and made pathological diagnosis of tumor. SM: guided treatment decisions and contributed to the management of patient care.
